# Efficacy of acupoint injection of recombinant human erythropoietin in the treatment of postoperative hemorrhagic anemia in elderly patients with femoral intertrochanteric fracture

**DOI:** 10.12669/pjms.41.12.13244

**Published:** 2025-12

**Authors:** Li Fan, Yuxia Jiang, Xiaohong Tao

**Affiliations:** 1 Li Fan, Department of Hematology, Zhejiang Provincial Tongde Hospital, Hangzhou, Zhejiang Province 310012, P.R. China; 2 Yuxia Jiang, Department of Hematology, Zhejiang Provincial Tongde Hospital, Hangzhou, Zhejiang Province 310012, P.R. China; 3 Xiaohong Tao, Department of Hematology, Zhejiang Provincial Tongde Hospital, Hangzhou, Zhejiang Province 310012, P.R. China

**Keywords:** Acupoint, Femoral intertrochanteric fracture, Hemorrhagic anemia, Injection, Recombinant human erythropoietin, Subcutaneous

## Abstract

**Objective:**

To evaluate the efficacy and safety of acupoint injection of recombinant human erythropoietin (rHuEPO) in the treatment of postoperative hemorrhagic anemia in elderly patients with femoral intertrochanteric fracture (FIF).

**Methodology:**

This retrospective cohort analysis was conducted at Zhejiang Provincial Tongde Hospital. The research data were obtained from 60 elderly patients with hemorrhagic anemia after FIF surgery who were treated from April 2022 to December 2023. Patients who received acupoint injection of rHuEPO (acupoint group) were matched with the cohort of patients who underwent conventional subcutaneous injection of rHuEPO (subcutaneous group) in a 1:1 ratio, 30 patients in each group. The postoperative clinical efficacy, levels of blood routine indicators, coagulation function, transfusion status, and incidence of adverse reactions were compared between the two groups.

**Results:**

The clinical efficacy in the acupoint group was higher than that in the subcutaneous group (P<0.05). One day after surgery, there was no significant difference in blood routine indicators between the two groups (P>0.05). Seven and 14 days after surgery, the levels of red blood cell (RBC), hemoglobin (Hb), and hematocrit (HCT) in both groups increased, and were higher in the acupoint group than in the subcutaneous group (P<0.05). There were significant time-dependent changes in coagulation parameters — prothrombin time (PT), activated partial thromboplastin time (APTT), and fibrinogen (FIB) — in both groups at 1, 7, and 14 days after surgery (P<0.05), without significant differences between the two groups (P>0.05). The blood transfusion in the acupoint group was lower than that in the subcutaneous group (P<0.05).

**Conclusions:**

Compared with the conventional subcutaneous injection, acupoint injection of rHuEPO seems to be more effective in treating elderly patients with hemorrhagic anemia after FIF surgery.

## INTRODUCTION

With the gradual aging of the population, the incidence of hip fractures is on the rise.^1^ In elderly patients, femoral intertrochanteric fracture (FIF), one of the common types of hip fractures, is associated with an almost 15% mortality due to various complications.^1^-^3^ Anemia occurs in 30-40% of hip fracture patients and is considered the most common complication associated with increased perioperative mortality.^4^,^5^ As FIF is an extracapsular fracture that is associated with more substantial blood loss compared to an intracapsular fracture, patients often have anemia before surgery.^1^,^2^,^5^ Additionally, elderly FIF patients are often treated with intramedullary nail fixation surgery, and intraoperative procedures such as marrow expansion can increase intraoperative and postoperative blood loss.^5^,^6^ Therefore, elderly patients with FIF experience more blood loss and severe anemia during the perioperative period, which requires greater attention to perioperative blood management, such as blood transfusion.^6^ The perioperative blood transfusion for elderly patients with hip fractures is as high as 70%.^6^ Blood products come from scarce sources, are expensive, and also face risks such as transfusion-related infections, immune reactions, cardiovascular dysfunction, and even death. Currently, guidelines suggest limited use of blood products.^4^-^6^

In recent years, recombinant human erythropoietin (rHuEPO) has been widely used in clinical practice, including fracture patients with perioperative anemia.^7^ rHuEPO, also known as red blood cell-stimulating factor or erythropoietin, is an endogenous glycoprotein hormone that can stimulate red blood cell production.^8^ At present, subcutaneous injections of rHuEPO are widely used to treat anemia associated with renal insufficiency, acquired immunodeficiency syndrome/AIDS, cancer, and rheumatism.^7^ The research has confirmed that the use of rHuEPO alone or in combination with iron can safely and effectively improve perioperative anemia in orthopedic surgery patients and reduce transfusion rates.^9^,^10^ However, the use of rHuEPO has certain limitations. A study showed that the typical response time of anemia patients to rHuEPO treatment is 2-4 weeks, the effective rate is only about 75%, and the costs are higher than blood transfusion.^11^ Therefore, improving the efficacy of rHuEPO is currently the main issue that needs to be addressed in clinical practice.

Acupoint injection technology is guided by the fundamental theories of traditional Chinese medicine (TCM) to stimulate the therapeutic effects of meridians and acupoints.^12^,^13^ It is a unique therapy formed by combining pharmacological effects and injection methods in modern medicine.^12^ An animal experiment showed that acupoint injection has a better anesthetic effect than subcutaneous injection.^13^ Li et al.^14^ also showed that compared with subcutaneous injection, acupoint injection can significantly improve cerebral hemodynamics and metabolism of cerebral nerve substances in children with cerebral palsy. This indicates that acupoint injection can exert better pharmacological effects.

At present, there are no reports on the treatment of postoperative anemia in elderly patients with FIF by acupoint injection of rHuEPO. In recent years, we have performed acupoint injection of rHuEPO treatment on some elderly patients with FIF. We This study aimed to evaluate the effectiveness of acupoint injection of rHuEPO in treating postoperative anemia in elderly patients with FIF.

## METHODOLOGY

This retrospective cohort analysis was conducted at Zhejiang Provincial Tongde Hospital. The research data were obtained from 60 elderly FIF patients who underwent intramedullary nail proximal femoral nail anti-rotation (PFNA) treatment from April 2022 to December 2023. Among them, 30 patients received acupoint injection of rHuEPO and were matched to the cohort of 30 patients who underwent conventional subcutaneous injection of rHuEPO.

### Ethical approval:

It was obtained for the Ethics Committee of Zhejiang Provincial Tongde Hospital reviewed and approved the study involving human participants. Approval number 2022085-JY; date: May 5, 2022. All patients provided written informed consent prior to treatment, including consent for receiving the assigned intervention and for the use of anonymized clinical data for research purposes.

### Inclusion Criteria:

- Meets the diagnostic criteria of FIF.
- Age ≥ 65 years old.
- Accept PFNA internal fixation surgery.
- Preoperative hemoglobin (Hb) level greater than 110g/L.
- No blood transfusion was performed during the operation.
- Complete clinical data, adhere to treatment of patients according to plan.

### Exclusion criteria:

- Bilateral fractures.
- Preoperative Hb is less than 90g/L.
- History of coagulation dysfunction, lower limb deep vein thrombosis, pulmonary embolism, myocardial infarction, cerebral infarction, etc.
- Preoperative use of anticoagulant drugs.
- Patients at high risk of thrombosis, including those with atrial fibrillation, pacemakers, and stent implantation.
- Combined hematopoietic system diseases and severe liver and kidney dysfunction.
- Merge fractures from other parts and pathological fractures.

### Intravenous infusion of sucrose iron treatment:

Starting from the first day after surgery, the patient is given sucrose iron injection (manufacturer: Nanjing Hengsheng Pharmaceutical Co., Ltd.) 100mg added to 100ml of sodium chloride solution by intravenous drip, once every other day, for a total of two weeks. If the patient’s Hb rises to 120 g/L and there is no iron overload after re-examination, medication can be continued until iron deficiency anemia and the condition are restored.

### Subcutaneous injection of rHuEPO:

rHuEPO (manufacturer: Shenyang Sansheng Pharmaceutical Co., Ltd.) is administered at a dose of 150U/kg each time, using a 2.5ml syringe with a standard subcutaneous injection needle to extract the approved dose of rHuEPO, and inject it according to the conventional subcutaneous injection method. The injection is administered three times per week for a total of two weeks. If the patient’s Hb is ≥ 120 g/L during the treatment process, rHuEPO can be discontinued.

### Acupoint injection of rHuEPO:

The patient’s bilateral blood points are selected in sequence and alternated as injection points. The dosage of rHuEPO is 150 U/kg each time, three times/week, for a total of two weeks. The method of acupoint injection is as follows: the patient is in a supine and flexed position. Using a 2.5-ml syringe with a standard subcutaneous injection needle, the approved dose of rHuEPO is extracted and vertically inserted into the Xuehai (SP10) acupoint, 1.0-1.5 inches. After inserting the needle, it is gently twisted and lifted until there is a feeling of soreness, swelling, or numbness in the local area. If no blood is drawn, the medication can be slowly injected. After withdrawing the needle, a sterile cotton ball is used to apply temporary pressure to prevent bleeding. During the treatment process, if the patient’s Hb is ≥ 120g/L, the use of rHuEPO should be stopped.

### Collected data:

- Basic clinical information of patients, including gender, age, body mass index (BMI), comorbidities, cause of injury, affected side, time between injury and surgery, duration of surgery, American Society of Anesthesiology (ASA) grading, preoperative Hb levels, and intraoperative blood loss.
- Clinical efficacy: The evaluation criteria are based on the “Diagnosis and Efficacy Standards for Hematological Diseases” 3rd edition.^3^ An increase in Hb by ≥ 20g/L compared to baseline is considered a significant effect. An increase in Hb by 10.0-19.9 g/L compared to the baseline is deemed effective. Other Hb changes are deemed invalid. Total efficacy (significant effect+effective)/total number.
- Blood routine tests on days one, seven, and 14 after surgery, including red blood cell (RBC), Hb, and hematocrit (HCT) levels.
- Coagulation function on days one, seven, and 14 after surgery, including prothrombin time (PT), activated partial thromboplastin time (APTT), and fibrinogen (FIB) levels.
- Blood transfusion-related side effects and complications.

### Statistical analysis:

SPSS/PC statistical software (version 26.0; IBM Corp, Armonk, NY, USA) was used for all analyses. Count data were presented in the form of n (%), and Fisher’s exact test was used for intergroup differences in gender, comorbidities, cause of injury, affected side, ASA grading, efficacy, and adverse reactions. Prior to statistical comparisons, the distribution of continuous variables such as hemoglobin (Hb), red blood cell count (RBC), and hematocrit (HCT) was evaluated using the Shapiro–Wilk or Kolmogorov–Smirnov tests, along with visual assessment via histograms and normal probability plots. Variables that followed a normal distribution were analyzed using independent sample t-tests or paired t-tests as appropriate. For variables that deviated from normality, Mann–Whitney U tests or Wilcoxon signed-rank tests were applied. These methods ensured that the selection of statistical tests was based on distributional assumptions. In addition, repeated measures ANOVA was used to evaluate within group changes in quantitative variables in time trends. The statistical significance was set to P<0.05. PRISM 8.0 software (GraphPad, San Diego, USA) was used to prepare charts.

## RESULTS

In this study, clinical data of 60 elderly patients with FIF were retrospectively analyzed. The cohort was divided into two groups in a 1:1 ratio, 30 patients in each group, and included 22 males and 38 females. The age range was 66-92 years, with an average age of 76.6 ± 7.3 years. Table-I shows that there was no statistically significant difference in the baseline information between the two groups (P>0.05). Fourteen days after surgery, the efficacy of the acupoint group was higher than that of the subcutaneous group (90.0% vs. 63.3%) (P<0.05) Table-II.

**Table-I:** Comparison of baseline data between two groups of patients.

Variables	Acupoint group (n=30)	Subcutaneous group (n=30)	χ^2^/t/Z	P
Female (yes), n(%)	20 (66.7)	18(60.0)	-	0.789*
Age (years), Mean±SD	75.17±6.9	78.0±7.6	-1.492	0.141
BMI(kg/m^2^), Mean±SD	21.5±2.6	22.3±2.8	-1.230	0.224
Diabetes (yes), n(%)	15 (50.0)	13 (43.3)	-	0.796*
Hypertension (yes), n(%)	13 (43.3)	11 (36.7)	-	0.792*
Cause of injury, n(%)			-	0.567*
Fall injury	20 (66.7)	23 (76.7)		
Traffic accident injury	10 (33.3)	7 (23.3)		
Affected side, n(%)			-	0.601*
Left	19 (63.3)	16 (53.3)		
Right	11 (36.7)	14 (46.7)		
The interval between fracture and surgery (day), M (IQR)	5 (3, 5)	4 (3, 5)	-1.228	0.219
Surgical duration (minutes), Mean±SD	73.5±13.5	77.7±10.4	-1.361	0.179
ASA grading, n(%)			-	0.842*
I	2 (6.7)	1 (3.3)		
II	14 (46.7)	16 (53.3)		
III	14 (46.7)	13 (43.4)		
Preoperative Hb level (g/L), Mean±SD	141.4±16.7	145.2±14.8	-0.932	0.355
Intraoperative blood loss (ml), Mean±SD	108.8±29.1	121.6±33.2	-1.584	0.119

Note:

* *P*, Fisher exact test

**Table-II:** Comparison of treatment efficacy between two groups.

Group	Significant effect	Effective	Invalid	Effective rate
Acupoint group (n=30)	25 (83.3)	2 (6.7)	3 (10.0)	27 (90.0)
Subcutaneous group (n=30)	11 (36.7)	8 (26.6)	11 (36.7)	19 (63.3)
*P				0.030

Note:

* *P*, Fisher exact test

**Fig.1:**
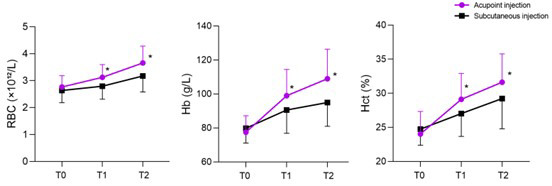
The change curves of RBC, Hb, and Hct levels in two groups. Compared with the subcutaneous group, * P<0.05. T0, 1-day after surgery; T1, 7-days after surgery; T2, 14-days after surgery. RBC, red blood cell; Hb, hemoglobin; HCT, hematocrit.

**Fig.2:**
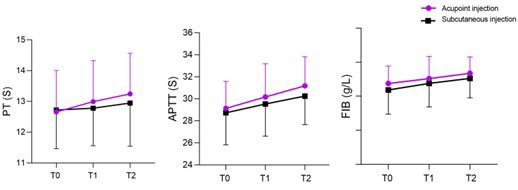
The change curves of PT, APTT, and FIB level for two groups. Compared with the subcutaneous group, * P<0.05. T0, 1-day after surgery; T1, 7-days after surgery; T2, 14-days after surgery. PT, prothrombin time; APTT, activated partial thromboplastin time; FIB, fibrinogen.

As shown in Fig.1, the levels of RBC, Hb, and Hct in both groups of patients increased over time (P<0.05). Seven and 14 days after surgery, the acupoint group demonstrated significantly higher levels of RBC, Hct, and Hb than the subcutaneous group (P<0.05). The levels of PT, APTT, and FIB in both groups changed significantly over time (Fig. 2; P<0.05), but there were no statistically significant differences between the two groups at any time point (P>0.05). The transfusion rate in the acupoint group was lower than that in the subcutaneous group (10.0% vs. 36.7%) (P<0.05). There was no statistically significant difference in the incidence of various complications between the two groups (P>0.05), Table-III. No local injection site-related complications such as erythema, hematoma, induration, or infection were observed in either group during hospitalization or documented in nursing follow-up records.

**Table-III:** Comparison of transfusion rates and complications between two groups.

Variables	Acupoint group (n=30)	Subcutaneous group (n=30)	*P
Transfusion rate, n(%)	3 (10.0)	11 (36.7)	0.030
Complications			
Gastrointestinal Discomfort	1 (3.3)	3 (10.0)	0.612
Dizzy	1 (3.3)	4 (13.3)	0.353
Constipation	1 (3.3)	3 (10.0)	0.612
Skin allergies	2 (6.7)	4 (13.3)	0.671
Pulmonary embolism	0 (0)	1 (3.3)	1.000
Deep vein thrombosis	0 (0)	1 (3.3)	1.000

Note:

* *P*, Fisher exact test

## DISCUSSION

To our knowledge, this is the first study comparing the efficacy and safety of subcutaneous and acupoint injection of rHuEPO in treating hemorrhagic anemia in elderly patients after FIF surgery. The results showed that compared with subcutaneous injection, acupoint injection of rHuEPO had a significant effect on the treatment of postoperative hemorrhagic anemia in elderly patients with FIF, with better blood routine levels and lower transfusion rates. This is consistent with the previous data on rHuEPO injections into relevant acupoints for the treatment of anemia.^15^,^16^ Our research results show that the clinical efficacy of rHuEPO acupoint injection is significantly higher than that of the subcutaneous one (90.0% vs. 63.3%). As a precursor cytokine for bone marrow red blood cells, endogenous erythropoietin is secreted by the kidneys, regulates the generation of red blood cells, and is essential for red blood cell production.^7^,^9^ The effectiveness of rHuEPO in treating perioperative anemia in fracture patients has been confirmed by relevant studies.^17^,^18^

The Xuehai (SP10) acupoint can promote the biochemistry of qi and blood by strengthening the spleen (at the Foot Tai Yin Spleen Meridian acupoint). Simultaneously, it stimulates blood circulation and disperses blood stasis to eliminate pathological products, while balancing “tonifying” and “promoting”, which is more in line with the complex state of “deficiency with blood stasis” in elderly patients.^12^,^19^ In this study, the Xuehai (SP10) was chosen as the injection point to achieve a combination of acupuncture and medication effects. Acupuncture and medication directly stimulate the acupoints along the meridians, producing specific therapeutic effects. At the same time, after acupoint injection, the medication remains at the acupoint for a more extended period of time.^20^ Therefore, it can enhance and prolong the therapeutic efficacy of acupoints, enabling them to follow and clear the meridians, and directly reach the corresponding pathological tissues and organs, fully exerting the joint therapeutic effect of acupoints and drugs.^20^,^21^

Increasing evidence supports that acupoint injection exerts its therapeutic effect not only through pharmacological action but also via modulation of the neuro-immuno-endocrine network.^22^ Stimulation of the SP10 (Xuehai) acupoint can activate sympathetic and vagal neural pathways, improve bone marrow microcirculation, and promote erythropoiesis.^23^ Moreover, acupoint injection induces cytokine release (e.g., IL-6, GM-CSF, and endogenous EPO), enhancing red cell precursor proliferation and differentiation. It may also activate the hypothalamic–pituitary–adrenal axis to promote endogenous erythropoietin secretion.^24^ These multi-level interactions between neural, immune, and endocrine systems may explain the superior hematopoietic efficacy observed in the acupoint injection group.

Conventional drugs can also act on acupoints through the nervous endocrine immune system, stimulating the body’s disease resistance and producing greater therapeutic effects.^20^,^21^,^25^ Studies have shown that acupoint injection therapy can deliver high doses of intravenous injection with equal or more potent efficacy in a short period at low doses.^25^,^26^ In this study, both groups used similar dosages and administration regimens. This allows for avoiding the influence of heterogeneity on the research results.

Acupoint injection has the characteristic of long-lasting efficacy; it is speculated that slowly absorbed drugs can continuously stimulate relevant acupoints and have similar effects as needling of specific acupoints.^25^-^27^ The results of this study showed that 14 days after surgery, the recovery rate was faster, and the changes in the levels of RBC, Hb, HCT, and other indicators were greater in the acupoint injection group. Moreover, the blood transfusion in the acupoint injection group was significantly lower than that in the subcutaneous injection group (10.0% vs. 36.7%). This effect may be due to the dual regulatory effect of “medication+acupoint” through acupoint injection, making it superior to subcutaneous injection in terms of clinical efficacy and improvement of blood routine indicators.^28^,^29^

In this study, both groups showed a significant increase in coagulation function indicators over time. However, there was no statistically significant difference between the two groups at each time point. It is possible that in the treatment of hemorrhagic anemia, the improvement of coagulation function relies more on the direct action of drugs. Moreover, the advantages of acupoint injection are not reflected in the regulation of coagulation indicators.^30^,^31^ There was no difference in the incidence of various complications between the two groups, indicating that acupoint injection therapy has the same safety. In summary, our research findings indicate that compared to subcutaneous injection, acupoint injection has higher benefits and is equally safe for treating postoperative hemorrhagic anemia in elderly FIF patients. The inferior hematologic outcomes observed in the subcutaneous injection group may be attributed to several physiological and pharmacokinetic factors, especially in elderly patients. Aging is associated with reduced subcutaneous tissue perfusion and decreased skin elasticity, which can impair drug diffusion and absorption, leading to lower bioavailability.^19^,^20^ Moreover, individual differences in injection site characteristics and systemic circulation may further increase variability in therapeutic response.^21^,^25^,^26^ In contrast, acupoint injection may prolong local drug retention and concurrently stimulate neural and immune pathways, potentially enhancing erythropoietic activity.^27^-^29^

### Strengths of the study:

This study has several strengths. To the best of our knowledge, few studies have directly compared the clinical efficacy of acupoint injection versus subcutaneous injection of rHuEPO in elderly patients with postoperative hemorrhagic anemia following intertrochanteric fracture. By applying strict inclusion and exclusion criteria along with a 1:1 matched design, we ensured a high degree of baseline comparability between groups. The findings provide preliminary evidence suggesting that integrative acupoint-based therapy may help reduce transfusion needs and accelerate early hemoglobin recovery in this patient population. Future research is warranted to determine whether these short-term benefits are sustainable over longer follow-up periods. In addition, upcoming studies should incorporate patient-reported outcomes such as functional recovery, fatigue, and quality of life, and adopt prospective, multicenter, randomized controlled trial designs. Mechanistic investigations are also needed to elucidate the neuro-immuno-endocrine pathways potentially underlying the observed effects.

### Limitations:

Firstly, it is a single-center retrospective analysis with a relatively small sample size (n=60), which may limit the statistical power, particularly for detecting differences in low-frequency secondary outcomes such as adverse events. Although a 1:1 matched design was employed based on key clinical variables (e.g., age, sex, ASA grade, and preoperative hemoglobin levels), residual confounding cannot be entirely excluded due to the absence of advanced causal inference methods such as propensity score matching (PSM) or inverse probability of treatment weighting (IPTW). Secondly, covariates such as preoperative hemoglobin and intraoperative blood loss, though balanced between groups and statistically non-significant, may still influence postoperative recovery. Given the limited sample size, we did not perform covariate-adjusted modeling (e.g., ANCOVA or multivariate regression) to avoid overfitting. Future studies with larger datasets are encouraged to apply such models for more precise adjustment. Thirdly, the study only evaluated hematologic outcomes within 14 days postoperatively. The long-term sustainability of hemoglobin improvement and the potential for anemia recurrence remain unclear. Follow-up data beyond 14 days were not available due to the retrospective nature of the study. Future prospective studies with extended follow-up (e.g., 30 to 90 days or more) are warranted to assess treatment durability and long-term safety. Fourth, although all patients received intravenous sucrose iron at a standardized dose (100 mg every other day for two weeks), this may have synergistically enhanced erythropoiesis, thereby confounding the isolated effect of injection route. The lack of variability in iron dosage precluded statistical adjustment using iron dose as a covariate. Future studies should consider iron-free control groups or incorporate iron-related variables into multivariable analyses. Fifth, this study did not assess patient-reported outcomes (PROs) such as functional recovery, quality of life, or fatigue. As a retrospective study based on electronic records, standardized instruments such as the Barthel Index, EQ-5D, or FACIT-F were unavailable. Nonetheless, hematologic markers such as hemoglobin and hematocrit are closely associated with physical recovery and may indirectly reflect functional status. Future prospective research should incorporate PRO measures to enhance the clinical relevance and translational value of acupoint injection. Finally, patient tolerance and local pain associated with acupoint injection were not assessed due to the absence of Visual Analog Scale (VAS) data or satisfaction surveys in the medical records. Future studies should include these measures to better evaluate tolerability and patient acceptance of acupoint versus subcutaneous injection.

## CONCLUSION

Our research results show that compared with subcutaneous injection, acupoint injection of rHuEPO seems to be more effective in treating postoperative hemorrhagic anemia in elderly FIF patients. However, the advantages of the method are not absolute, and personalized plans need to be developed by professionals based on the specific conditions of the patient (such as anemia level, physical condition, sensitivity to acupoint stimulation, etc.).

### Authors’ contributions:

**LF:** Study design, literature search and manuscript writing.

**YJ and XT:** Data collection, data analysis and interpretation. Critical Review.

**LF:** Manuscript revision and validation and is responsible for the integrity of the study.

All authors have read and approved the final manuscript.
